# Upper airway stabilization by osteopathic manipulation of the sphenopalatine ganglion versus sham manipulation in OSAS patients: a proof-of-concept, randomized, crossover, double-blind, controlled study

**DOI:** 10.1186/s12906-017-2053-0

**Published:** 2017-12-20

**Authors:** Olivier Jacq, Isabelle Arnulf, Thomas Similowski, Valérie Attali

**Affiliations:** 1 Sorbonne Universités, UPMC Université Paris 06, INSERM, UMRS1158 Neurophysiologie Respiratoire Expérimentale et Clinique, Paris, France; 2 AP-HP, Groupe Hospitalier Pitié-Salpêtrière Charles Foix, Service d’Exploration des Pathologies du Sommeil (Département “R3S”), 47-83 boulevard de l’hôpital, 75013 Paris, France; 3 Department of Sleep Medicine (“Service des Pathologies du Sommeil”), Pitié-Salpêtrière Hospital, 47-83 Bd de l’Hôpital, 75651 Paris Cedex 13, France

**Keywords:** Obstructive sleep apnoea, Osteopathic manipulative treatment, Intraoral manipulation, Sphenopalatine ganglion, Critical closing pressure

## Abstract

**Background:**

Osteopathic manipulative treatment (OMT) of the sphenopalatine ganglion (SPG) is used empirically for the treatment of rhinitis and snoring and is thought to increase pharyngeal stability. This trial was designed to study the effects of this treatment on pharyngeal stability evaluated by critical closing pressure in obstructive sleep apnoea syndrome.

**Methods:**

This single-centre, randomized, crossover, double-blind study compared active manipulation and sham manipulation of the SPG. Randomization was computer-generated. Patients each received one active manipulation and one sham manipulation at an interval of 21 days and were evaluated 30 min and 48 h after each session administered by a qualified osteopath. Neither the patients, nor the investigator performing the evaluations were informed about the order of the two techniques (double-blind). The primary endpoint was the percentage of responding patients presenting increased pharyngeal stability defined by a variation of critical closing pressure (Pcrit) of at least −4 cmH_2_O at 30 min. Secondary endpoints were the variation of Pcrit in absolute values, sleepiness and snoring. Others endpoints were lacrimation (Schirmer’s test), induced pain, sensations experienced during OMT.

**Results:**

Ten patients were included and nine (57 [50; 58] years, comprising 7 men, with an apnoea-hypopnoea index of 31.0 [25.5; 33.2]/h; (values are median [quartiles])) were analysed. Seven patients were analysed for the primary endpoint and nine patients were analysed for secondary endpoints. Five patients responded after active manipulation versus no patients after sham manipulation (*p* = 0.0209). Active manipulation induced more intense pain (*p* = 0.0089), increased lacrimation (ns) and more tactile, nociceptive and gustatory sensations (13 versus 1) compared to sham manipulation. No significant difference was observed for the other endpoints.

**Conclusions:**

Osteopathic manipulative treatment of the SPG may improve pharyngeal stability in obstructive sleep apnoea syndrome. This trial validates the feasibility of the randomized, controlled, double-blind methodology for evaluation of this osteopathic treatment. Studies on a larger sample size must specify the efficacy on the apnoea-hypopnoea index.

**Trial registration:**

The study was retrospectively registered in the clinicaltrial.gov registry under reference NCT01193738 on 1st September 2010 (first inclusion May 19, 2010).

**Electronic supplementary material:**

The online version of this article (doi: 10.1186/s12906-017-2053-0) contains supplementary material, which is available to authorized users.

## Background

Obstructive sleep apnoea syndrome (OSAS) is characterized by repeated upper airway obstruction during sleep, which induces interruption of ventilation, intermittent desaturation, microarousals and a transient increase of sympathetic tone [1]. Severe OSAS is responsible for accidents related to excessive daytime sleepiness [2] and cardiovascular [3], cognitive [4] and metabolic [5] consequences. Nocturnal continuous positive airway pressure (CPAP) ventilation [3], the reference treatment, and mandibular advancement devices, the most frequent alternative treatment in patients not supporting CPAP [6, 7], prevent obstructive events by modifying upper airway anatomy, to enlarge and maintain an open airway. However, although obstructive apnoeas often have an anatomical origin (excessively narrow upper airways, macroglossia) [8], more than 50% of patients experience obstructive apnoeas with no major anatomical abnormality [9]. This suggests that functional abnormalities of the upper airways also contribute to the pathophysiology of OSAS. The maintenance of upper airway patency throughout the respiratory cycle is dependent of upper airways dilator muscles, which are mainly innervated by the hypoglossal nerve [10]. In OSAS patients, these muscles comprise a smaller proportion of type I muscle fibres [11] and present reduced metabolic activity [12]. Moreover, alteration of the neural control of upper aiways are also present in OSA patients: peripheral sensory neuropathy [13], hypoglossal motor neuropathy [14], and abnormal respiratory-related cortical adaptations [15] have been reported. These abnormalities may contribute to obstructive events during sleep by promoting upper airways unstability [16]. This probably explains why “functional” treatment, such as pharyngeal muscle retraining which acts via a combination of increased strength of the genioglossus, the main dilator muscle of the upper airways, and neuromodulating adaptations [17], has been shown to effectively reduce the apnoea-hypopnoea index (AHI) [18].

The sphenopalatine ganglion (SPG) is an autonomic nervous system ganglion that relays mixed cranial nerves innervating the upper airways. The SPG is situated in the pterygopalatine fossa, posterior to the posterior wall of the maxillary sinus and inferior to the junction of the body of the sphenoid, the greater wing and pterygoid process of the sphenoid, lateral to the perpendicular plate of the palatine bone, and medial to the pterygomaxillary fissure. It receives parasympathetic and sympathetic sensory afferents via fibres derived from the accessory branch of the facial nerve (VIIb) and the maxillary branch of the trigeminal nerve (V2). It distributes these fibres to the nasal mucosa, lacrimal glands, nasopharynx and soft palate, including some of the upper airway dilator muscles [19]. It could therefore play an important role in control of upper airway stability, by modulating nasal congestion, and/or upper airways muscles tone throughout the respiratory cycle. The SPG is targeted in the treatment of cluster headache, based on the fact that parasympathetic hyperactivity plays an important role in its physiopathology [20]. Postganglionic parasympathetic blockade of the SPG by local anaesthesia or implanted stimulation alleviates pain and nasal congestion in cluster headache [20, 21].

Intraoral myofascial therapy of the SPG is widely used in osteopathic practice, for the management of nasal obstruction, chronic rhinitis and snoring [22]. It is possible that this therapy allows to obtain muscle relaxation and to relieve pain in patients with temporomandibular dysfunction [23]. Clinical experience and upublished observations also suggest reduction of snoring (reflecting upper airway instability during sleep) after osteopathic manipulative treatment (OMT) of the SPG. On the basis of the above reasoning, weakly supported by unpublished reports from practitionners, it seemed interesting to test the hypothesis that OMT of the SPG could improve upper airway stability in OSAS patients. We designed a randomized, crossover, double-blind, controlled (active manipulation vs. sham manipulation) proof-of-concept trial, in which the primary endpoint was determination of upper airway critical closing pressure in awake subjects (Pcrit; defined as the negative pressure beyond which the upper airways collapse, and recognized as an index of upper airway collapsibility [24]). This study was retrospectively registered in the clinicaltrial.gov registry on 1st September 2010 under reference NCT01193738. The first patient was included on 19 May 2010 and the last visit of last patient was done on 25 May 2011. The trial ended when the needed number of patients was reached. The results were presented in an oral communication to the congress of the *Société Française de Médecine du Sommeil* (SFRMS) [(French society of sleep medicine] in November 2015.

## Methods

### Study design

**Fig. 1:**
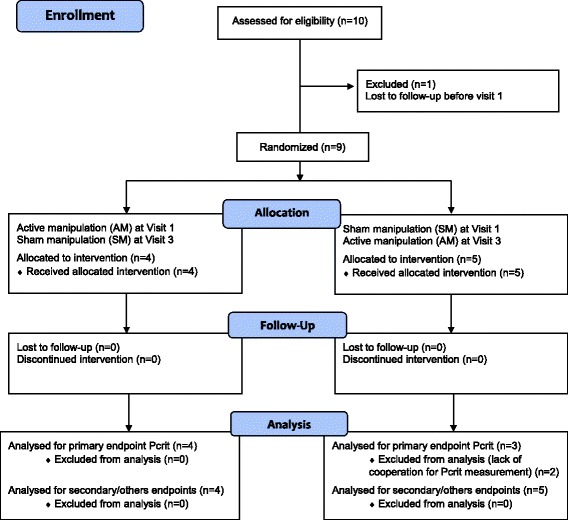
Flow chart

This randomized, controlled, crossover, double-blind study compared osteopathic active manipulation (AM) and sham manipulation (SM) of the SPG. Patients were randomized to receive either AM first, followed by SM 21 days later, or vice versa. The protocol comprised four visits. Active or sham manipulations were performed at visits 1 and 3. Visit 2 was held 48 h after visit 1 and visit 4 was held 48 h after visit 3. The effect of treatment was evaluated 30 min after AM or SM at visits 1 and 3 and 48 h after AM or SM at visits 2 and 4. The study flow chart is presented in Fig. 1. This study was approved by the *Comittee for the Protection of Human Research Participants, Paris VI (Comité de Protection des Personnes Ile-de-France VI, Paris, France*) (IEC/IRB). All patients signed an informed consent form.

### Patients

Patients included in this study were 18 years or older, with OSAS and an AHI ≥ 15/h and ≤ 45/h, recruited in a specialized centre (Department of Sleep Medicine -Department R3S, Pitié-Salpêtrière-Charles Foix Hospital Group, Paris). Exclusion criteria were as follows: patients treated by nocturnal continuous positive airway pressure or mandibular advancement devices unable to temporarily stop this treatment for the purposes of the study, or presenting complete nasal obstruction; patients treated with serotonin reuptake inhibitors or with a BMI > 40 kg/m^2^. Patients previously treated for their OSAS by CPAP or mandibular advancement devices had to stop their treatment 1 week before visit 1, then transiently resumed their treatment after visit 2 and had to stop treatment again 1 week before visit 3 and resume treatment after visit 4.

### Interventions

Active osteopathic manipulation (AM) and sham manipulation (SM) consisted of purely manual pressure, administered by a single qualified osteopath for all patients.

**Fig. 2:**
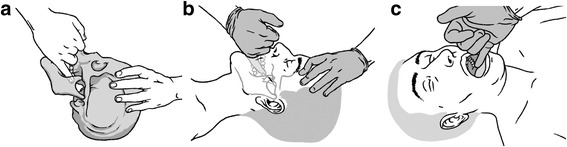
**a**, **b**, **c** Intraoral myofascial therapy of the sphenopalatine ganglion. Position of the patient’s head and the osteopath’s fifth finger, during SPG release

AM: Pressure was applied to the left and right SPGs successively. The method used complies with the description by Kalamir et al. [23]. The patient was placed in the supine position. The osteopath wore gloves and was seated next to the patient on the opposite side to the first SPG to be treated. One of the osteopath’s hands was placed with the palm open in contact with the patient’s vertex to stabilize the patient’s head. The patient was asked to open the mouth and shift the mandible laterally towards the side of the SPG to be treated. The osteopath then applied pressure to the SPG with the fifth finger of his free hand in the patient’s mouth, ascending along the alveolar process of the maxilla to reach the pterygoid process. The osteopath then ascended his finger cephalically and medially towards the SPG, and then immobilized his hand until relaxation of the external pterygoid muscle (about 15 s) before advancing medially and cephalically into the pterygopalatine fossa as far as the SPG. The osteopath then exerted light pressure on the SPG with the pulp of his fifth finger until release of the tissues. He then performed contralateral SPG release according to the same technique. Figure 2a, b, c shows the position of the patient’s head and the osteopath’s fifth finger, during SPG release.

SM: The osteopath and patient were in the same respective positions as for AM and the same instructions were given to the patient. SM was performed with an intraoral gloved hand, with the osteopath’s fifth finger placed laterally to the last homolateral molar without reaching the pterygoid process. With the fifth finger, the osteopath then successively applied repeated pressure on the adjacent oral mucosa. The duration of administration of SM was identical to that of AM.

### Outcome measures

#### Primary endpoint: waking Pcrit

The primary endpoint was the percentage of responding patients presenting increased pharyngeal stability defined by a variation of critical closing pressure (Pcrit) of at least −4 cmH_2_O at 30 min. The pressure/flow relationship was established to measure the critical closing pressure, Pcrit, defined as the negative pressure inducing upper airway collapse (absence of flow). Pcrit was determined while awake, according to the method validated by Su et al. [25]. Briefly, the patient was installed in the supine position with the head resting in a neutral position on a flat pillow. The position of the head was maintained with a foam collar. A nasal mask was applied and connected to a circuit allowing the generation of increasing negative pressures. Flow and pressure in the mask were measured through a mouthpiece and a pneumotachograph (Hans Rudolph Model 4700; Hans Rudolph, Inc., Kansas City, MO). Pcrit was then estimated by linear regression by reporting pressure and flow values at each imposed pressure phase. Pcrit was measured at baseline, 30 min and 48 h after administration of AM or SM.

#### Secondary endpoints

The variation of Pcrit in absolute values after 30 min and after 48 h, and the percentage of responding patients presenting increased pharyngeal stability defined by a variation of critical closing pressure (Pcrit) of at least −4 cmH_2_O, at 48 h were analysed as secondary endpoints. Sleepiness was evaluated by Epworth sleepiness scale [26]. Snoring was evaluated by clinical interview of the partner and concerned the two nights preceding each visit (“*According to your partner, did you snore during the previous two nights?”).* At visits 2 and 4, the patient was also asked whether snoring had increased, decreased or stayed the same compared to visit 1 (for visit 2) or compared to visit 3 (for visit 4).

#### Others endpoints

*Evaluation of the pain induced* by AM and SM was performed by using a non-graduated 10 cm pain visual analogue scale (VAS) (no pain - worst imaginable pain), administered immediately after AM and SM.

*Evaluation of lacrimation induced by* AM and SM was performed using Schirmer’s test according to a validated method [27] during administration of AM and SM. Schirmer’s test consists of placing a calibrated strip of blotting paper in the lower conjunctival fornix of both eyes to absorb tears, and then measuring the length of moistened blotting paper. Strips of blotting paper were placed immediately before the first osteopathic manipulation of the SPG (AM or SM) and the duration of pressure applied to the SPG was measured. This procedure was repeated before manipulation of the contralateral SPG. The results are expressed in mm/s and correspond to the sum (right eye + left eye).

*Sensations induced by AM and SM* were evaluated by directive interview and free description of all sensations experienced, administered during the 30 min following AM or SM. Directive interview comprised the following questions: “*Did you experience any gustatory sensations (taste in the mouth), olfactory sensations (prominent odour), visual sensations (impression of bright light or darkening of the room), auditory sensations (sounds, blocked ear, loss of balance, ringing in the ears), tactile sensations (passage of fluid in the cheek, presence of a bruise, or other sensations), nociceptive sensations (pricking, electric shock or other sensations)?”* The patient was asked to simply answer “Yes” or “No” to each item and was then asked to freely describe all sensations experienced using his or her own words (free verbatim).

### Randomization and blinding

The order of administration of AM and SM was randomized. The random sequence was generated using the R statistical software [28]. It consisted in a single block of ten (five allocations to AM at visit 1 then SM at visit 3 and five allocations to SM at visit 1 then AM at visit 3). The allocation concealment was secured as follows. A research nurse not participating in the study itself was responsible for the random sequence generation and for treatment allocation. After the physician responsible for the clinical investigation (“investigator”) had confirmed the eligibility and collected baseline data of a given patient during visit 1, the research nurse provided the osteopath performing the AM and SM maneuver with this patient’s allocation, outside the knowledge of the investigator. The osteopath was himself ignorant of the randomization list as a whole. The investigator was not present when the osteopath performed the maneuvers, and was therefore blinded to the allocation when subsequently analyzing the data. As a result, both the patient and the investigator analyzing the data were blinded to the allocation. The osteopath was not involved in any manner in the data analysis. At the end of the study and to check a possible de-blindind, the participants were asked to indicate which of the two manipulations they believed was the active one.

### Sample size and statistical methods

The primary endpoint was the percentage of patients considered as responders, as follows. A patient was considered to be a responder to AM or SM in terms of the primary endpoint when Pcrit was decreased by at least −4 cmH_2_O (more negative values correspond to greater upper airway stability) at 30 min [29, 30]. As the variation of Pcrit after AM has never been previously evaluated, we considered a percentage of patients responders of 60% after AM and of 10% after SM. On this basis, complete data for six patients would be sufficient to demonstrate a difference between AM and SM with a type I error α = 0.05 and a power of 90%. By taking into account patients lost to follow-up and uninterpretable data, we considered that a sample of nine patients would be sufficient to demonstrate a difference between the two groups. Data analysis was performed by the blinded investigating physician after anonymization by patient and by visit. Statistical analysis was performed by using university on-line resources (biostatgv.sentiweb.fr). Data are expressed as median and quartiles. Continuous variables were compared by Mann-Whitney test and proportions were compared by Fisher’s exact test. The variation of snoring between visits 1 and 2 and between visits 1 and 3 was analysed by McNemar’s test.

## Results

Ten patients were included and nine patients completed all study visits (Fig. 1). The tenth patient consented to participate, and the first visit was planned a few days later, but he was lost to follow-up before this screening visit. Insofar as there was no baseline evaluation in this case, this patient does not appear in the analysed population. Patients 2, 3, 5 and 6, received active manipulation (AM) at visit 1 and sham manipulation (SM) at visit 3. Patients 1, 4, 7, 8 and 9 received SM at visit 1 and AM at visit 3. Table 1 presents the baseline characteristics before AM and SM. No significant difference was observed between the AM and SM groups in terms of these baseline characteristics.

**Table 1:** Baseline characteristics

*N* = 9			
Age (years)	57 [50; 58]		
Gender	7 M/2F		
Time since diagnosis (months)	13 [2; 17]		
OSAS treatment	CPAP =5; Oral appliance =1; none = 3		
AHI (number/h)	31.0 [25.5; 33.2]		
AI (number/h)	16.9 [10.8; 19.2]		
Time at SpO_2_ < 90% (%)	2.8 [0.4; 5.4]		
	Before AM	Before SM	p
BMI (kg/m^2^)	26.2 [24.8; 28.6]	27.0 [25.3; 29.1]	0.29
Neck circumference (cm)	40 [33; 42]	41 [33; 42]	0.93
Epworth sleepiness score (/24)	9 [5; 15]	6 [5; 9]	0.28
Snoring (yes/no/unknown)	5/2/2	6/1/2	0.81
Pcrit^a^ (cm H_2_O)	−21.0 [−26.7; −18.7]	−26.6 [−37.0; −20.2]	0.38
Schirmer’s test (left + right; mm/min)	33 [22; 53]	40 [19; 50]	0.82

*OSAS* Obstructive Sleep Apnea Syndrome, *CPAP* Continuous Positive Airway Pressure, *AHI* Apnea Hypopnea Index, *AI* Apnea Index, *AM* Active manipulation, *SM* Sham manipulation, *BMI* Body Mass Index, *Pcrit* Critical Closing Pressure

^a^ *n* = 7; Values are median [quartiles] or number of patients for gender, OSAS treatment and snoring

### Pcrit

Interpretable data were available for all visits in seven of the nine patients. No valid Pcrit measurement was available for patients 8 and 9 due to poor cooperation. The number of responding patients at 30 min (primary endpoint) was significantly higher after AM (5/7) than after SM (0/7) (*p* = 0.0209).

Relative variations of Pcrit, at 30 min, after AM (Pcrit_AM30_) and SM (Pcrit_SM30_) were −4.5[−9.9;-1.0] (*p* = 0.078) cmH_2_O and +1.7 [0.4; 6.5] cmH_2_O (*p* = 0.16), respectively. The median Pcrit_AM30_-Pcrit_SM30_ difference was −6.2[−22.0;-2.3] cmH_2_O (*p* = 0.078) (mean difference: −16.9 ± 25.2 cmH_2_O). At 48 h, 4 out of 7 patients were responders after AM versus only one patient after SM (*p* = 0.266). Three of the responders 30 min after AM were still responders at 48 h. Variations of Pcrit after AM (Pcrit_AM48_) and SM (Pcrit_SM48_) were −9.2[−20.9;-1.8] (*p* = 0.031) cmH_2_O and +1.9[−3.3; 12.4] cmH_2_O (*p* = 0.81), respectively. The median Pcrit_AM48_-Pcrit_SM48_ difference was −26[−32.9;-0.4] cmH_2_O (*p* = 0.109).

Table 2 presents the number of responding patients 30 min after each manipulation with the comparison between AM and SM at 30 min and 48 h. An additional table presents the individual data of Pcrit (see Additional file 1).

**Table 2:** Number of responding patients at 30 min and 48 h

*N* = 7	AM	SM	p
30 min	5	0	0.0209
48 h	4	1	0.266

*AM* Active manipulation, *SM* Sham manipulation

**Fig. 3:**
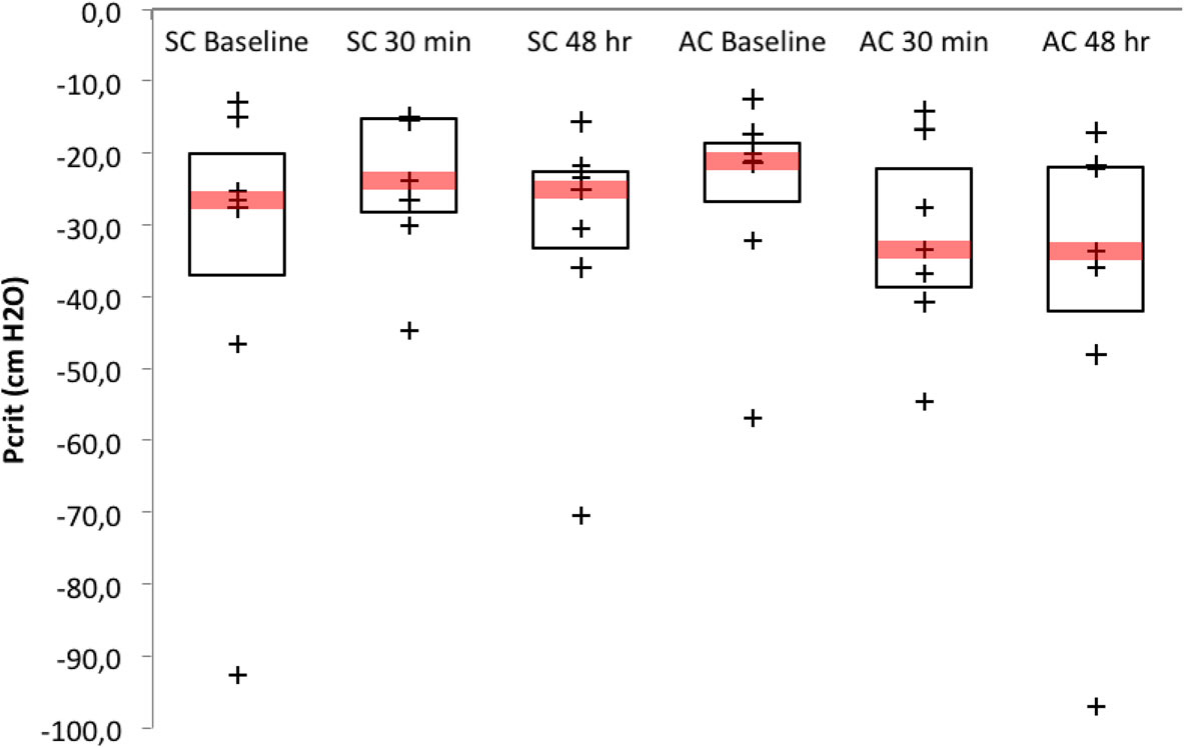
Upper airway critical closing pressure (Pcrit) before, 30 min and 48 h after sham manipulation (SM) (*left*) and active manipulation (AM) (*right*) of the sphenopalatine ganglion. The box represents the Q1-Q3 interquartile range, in which Q1 represents the first quartile and Q3 represents the third quartile. The bar in the *box* represents the median. The endpoint of the *lower* whisker is the minimum value higher than the *lower* limit defined by the following formula: Q1-1.5* (Q3-Q1). The endpoint of the *upper* whisker is the maximum value *lower* than the upper limit defined by the following formula: Q3 + 1.5* (Q3-Q1). + represent individual values

Figure 3 presents the variations of Pcrit 30 min and 48 h after SM and AM, respectively.

### Secondary endpoints (others than Pcrit)

Data for secondary endpoints were obtained at all visits in nine patients.

*Sleepiness*: The Epworth score decreased by −3[−5; 0] 48 h after AM and by −1[−1; 0] 48 h after SM; (AM-SM difference = −2[−4; 2]; *p* = 0.50).

*Snoring*: Before AM, six patients reported that they were snorers with snoring louder than speech, with missing data for three patients. Forty-eight hours after AM, three of the six snorers reported decreased intensity of snoring and three patients did not observe any difference (*p* = 0.24). Before SM, five patients reported that they were snorers with snoring louder than speech, with missing data for four patients. Forty-eight hours after SM, none of the five snorers reported any improvement and one patient reported more intense snoring (*p* = 1).

### Others endpoints

Data for others endpoints were obtained at all visits in nine patients.

*Pain induced* by AM and SM (VAS): All patients described sharp but tolerable and very brief pain during AM, and did not report any pain after SM. The median pain score on the visual analogue scale was 8.0 [6.0; 8.0] after AM and 0.0 [0.0; 1.0] after SM (difference: 6.5 [5.0; 8.0]; *p* = 0.0089).

*Lacrimation*: Lacrimation increased by 11[−11; 24] mm/s after AM and decreased by −8[−10; 6] mm/s after SM (AM-SM difference = 10[−3; 22]; ns). Five patients presented increased lacrimation, one patient presented identical lacrimation and three patients presented decreased lacrimation after AM. Three patients presented increased lacrimation and six patients presented decreased lacrimation after SM.

*Sensation questionnaire*: Patients reported significantly more sensations after AM than after SM, independently of the randomized order of administration. Patients reported nociceptive (*n* = 8), tactile (*n* = 4) and gustatory (*n* = 1) sensations after AM. No patient reported any visual or auditory sensations. Only one patient reported tactile sensations, in the absence of any other sensations, after SM. Analysis of verbatim descriptions revealed marked differences in the sensations experienced by patients between AM and SM. After AM, patients described a reduction of nasal congestion and/or a feeling of breathing more easily through the nose (*n* = 2), a feeling of mouth opening (*n* = 3), brief paraesthesia of the face (*n* = 5), a taste of blood in the mouth with no apparent bleeding (*n* = 1), a feeling of relaxation or release (*n* = 4) and fatigue (*n* = 1). After SM, patients reported a feeling of pressure or vibration on the gum (*n* = 4), jaw popping (*n* = 1), a feeling of mouth opening (*n* = 1) and increased salivation (*n* = 1). An additional table presents the patients’ verbatim descriptions after AM and SM (see Additional file 2). One patient reported resolution of latent headache present for 6 months.

*Answer to the question: “Which manipulation was the active one?”*: six out of 9 patients reported beint absolutely certain to have identified the active maneuver, but, they were all wrong. The three remaining patients answered that they were not able to determine which of the two manipulations was the active one.

An additional table presents the individual data of pain VAS, lacrimation, epworth and snoring, after AM and SM (see Additional file 1).

## Discussion

This is the first study to demonstrate the effect of osteopathic manipulation of the SPG on the stability of the upper airways in awake patients evaluated by a physiological endpoint (upper airway critical closing pressure). More generally, this is the first study to demonstrate the effect of osteopathic manipulative treatment on this endpoint.

### Methodological considerations

In the absence of any possibility to blind the osteopath performing the maneuver, we adopted a strategy derived from the “PROBE” concept (Prospective, Randomized, Open, Blinded, End-Point) [31], consisting of assessment of the endpoints by an investigator blinded to the nature of the intervention. However, in contrast to the PROBE design, we kept the patients blind to the treatment received. To this aim, particular attention was paid to the sham technique (same position of the patient and the osteopath, same instructions, same position of the osteopath’s hand, same duration). A significant difference was observed between AM and SM in terms of the pain induced by the intervention, but in the end, debriefing showed that 3 patients could not decide which manipulation was the active one and that the 6 who thought that they had identified the active manipulation were wrong. We therefore believe that the blinding of the patients was successful (without deblinding) and, as a result, that this randomized crossover controlled trial can be considered double-blind (patients and endpoint allocators). It complies with current guidelines (CONSORT) [32] (see Additional file 3). We acknoledge that ideally the study should have been triple-blinded (Osteopath, Investigator, Patient), but due to the nature of the intervention (manual therapy), it was not possible to blind the Osteopath. Although triple-blind could not be achieved, we submit that the design of the study constitutes its major strong point. Of note, based on the clinical experience of osteopaths performing SPG manipulation, a difference was also expected in terms of the lacrimation induced by the two interventions. However, only a non-significant trend towards a difference in lacrimation was observed (more marked lacrimation after AM).

Pcrit was used as the primary endpoint. Pcrit is a validated index of upper airway collapsibility [24], which has already been used in studies on OSAS, either during sleep [33] or in awake patients [25] in pathophysiology studies or to evaluate the efficacy of treatment [25, 34]. In our study, we decided to evaluate Pcrit in awake patients for obvious practical reasons. Consequently, the results of this study cannot be extrapolated to sleep and further studies therefore need to be conducted in sleeping patients. Nevertheless, the choice of Pcrit in awake patients as the primary efficacy endpoint of osteopathic manipulation of the SPG in the context of a pilot study appears to be justified in view of the known abnormalities of upper airway stability in awake patients with OSAS [29], and the known abnormalities of ventilatory control (see introduction) [15, 35, 36]. Pcrit measured in awake patients has been shown to be correlated with the contractile and metabolic properties of upper airway muscles [37]: it therefore appears reasonable to consider that Pcrit could constitute a relevant index to test the effect of an intervention with a presumed neuromodulation mechanism. The cut-off of −4 cm H_2_O used to define responding patients in this study is consistent with various data reported in the literature. For example, a study conducted in 54 OSAS patients showed that a − 3.0 cm H_2_O variation of Pcrit during sleep was sufficient to confirm improvement of upper airway stability in response to a therapeutic intervention [30]. A -4 cm H_2_O variation of Pcrit in awake patients corresponds to a sufficient increase of upper airway stability to avoid the obstructive events on inspiration [29], as, at each inspiration, the diaphragm produces a negative pressure that tends to close the upper airways and generate obstructive events when it is not counterbalanced by contraction of upper airway dilator muscles, mainly genioglossus [38]. Increased diameter [39] and greater stability of the upper airways [29] are therefore observed during inspiration.

### Clinical and pathophysiological considerations

A significantly higher proportion of patients experienced a − 4 cm H_2_O improvement of Pcrit at 30 min after AM than after SM, reflecting a significant effect of AM on upper airway stability. However, the difference in relative variation of Pcrit between AM and SM was not significant due to a lack of power induced by the marked interindividual variability, as a total of 66 subjects would have been necessary under two-sided conditions, with a power of 90% and a type I error of 0.05 to demonstrate a significant difference between the two groups on the basis of the values observed. Future studies will therefore need to determine the characteristics of responding patients.

In several patients, AM unblocked the nose and induced a series of sensory and somatosensory sensations, not observed after SM, suggesting autonomic neuromodulation, possibly via postganglionic parasympathetic blockade [19], as similar features have been described during implanted electrical stimulation of the SPG, used for the treatment of cluster headache [40, 41]. One patient in our study reported resolution of latent headache present for 6 months, thereby supporting the hypothesis of a similar type of neuromodulation mechanism induced by osteopathic manipulation of the SPG. The target of action of OMT could prove to be the proximal pharynx, as several patients reported easier nose breathing after AM, and the proximal pharynx corresponds to the zone of distribution of nerve fibres derived from the SPG. It could be useful to measure nasal obstruction by nasal peak flow meter in a subsequent study.

### Limitations

Despite the small sample size, this study identified a possible effect of a single osteopathic manipulation of the SPG on upper airway stability in awake patients, but does not justify any conclusions concerning the efficacy of this intervention in the treatment of OSAS. These preliminary results need to be confirmed by studies based on larger sample sizes and comprising evaluation of the apnoea-hypopnoea index. The choice to study the effect of a single osteopathic technique and to apply identical treatment to all patients allowed us to precisely identify the effects of this technique by minimizing possible biases. We are aware that this approach does not constitute the classical approach in osteopathic manipulative treatment, in which treatment is generally adapted to the patient’s needs and a combination of several techniques is usually proposed. This study suggests that osteopathic manipulation of the SPG induces a neuromodulation effect and improves the stability of the pharynx, but the mechanism of action and the duration of the effects produced cannot be more precisely defined. Although inhibition of postganglionic parasympathetic activity by osteopathic manipulation of the SPG appears likely, especially in view of resolution of blocked nose, modulation of sympathetic activity cannot be excluded in view of the increased lacrimation observed in some patients. The duration of the effect of OMT could not be determined in the present study. Nevertheless, a free interval of 3 weeks appeared to be sufficient to ensure the absence of a carryover effect. Finally, this study suggest the existence of responding patients, but their precise profile could not be identified.

## Conclusions

This study validates the feasibility of randomized, double-blind, controlled studies to evaluate the efficacy of osteopathic manipulation of the SPG in OSAS patients, particularly by validation of a sham osteopathic manipulation technique. These results provide the proof of concept of a significant effect of osteopathic manipulation of the SPG on upper airway stability in OSAS patients. Certain elements (relief of nasal obstruction, lacrimation, somatosensory sensations) suggest a neuromodulation mechanism of action of osteopathic manipulation of the SPG on the pharyngeal region. These data must be confirmed by a study on a larger sample size of patients, based on a more specific efficacy endpoint of OSAS, such as the apnoea-hypopnoea index.

## Additional files

Additional file 1: “additional table: individual data”. Individual data of Pcrit, pain VAS, lacrimation, epworth and snoring, after AM and SM. (DOCX 24 kb)

Additional file 2: “additional table: verbatim”. Patients’ verbatim descriptions after AM and SM, derivated from the sensation questionnaire. (DOCX 17 kb)

Additional file 3: “CONSORT 2010 checklist of information to include when reporting a randomised trial”. Checklist of information and page numbers where these informations can be found. (DOC 217 kb)

## Abbreviations

AHI: Apnoea-hypopnoea index
AM: Active manipulation
CPAP: Continuous positive airway pressure
MAD: Mandibular advancement device
OSAS: Obstructive sleep apnoea syndrome
Pcrit: Upper airway critical closing pressure
SM: Sham manipulation
SPG: Sphenopalatine ganglion
