# Supplementary material for: Upper airway stabilization by osteopathic manipulation of the sphenopalatine ganglion versus sham manipulation in OSAS patients: a proof-of-concept, randomized, crossover, double-blind, controlled study
Source: BMC Complement Altern Med. 2017 Dec 20;17:546. doi: 10.1186/s12906-017-2053-0 (PMC5738827; doi:10.1186/s12906-017-2053-0)
Supplement: Supplementary file 1 — “additional table: individual data”. Individual data of Pcrit, pain VAS, lacrimation, epworth and snoring, after AM and SM. (DOCX 24 kb) [file 12906_2017_2053_MOESM1_ESM.docx]

| **patient** | **gender** | **AHI (/hour)** |
| --- | --- | --- |
| 1 | Male | 31,9 |
| 2 | Male | 17,2 |
| 3 | Male | 24 |
| 4 | Female | 45,2 |
| 5 | Male | 47,4 |
| 6 | Male | 25,5 |
| 7 | Male | 31 |
| 8 | Male | 30,4 |
| 9 | Female | 33,2 |

Additional file 1

Additional table: Individual data

| **patient** | **Sham Manipulation** | | | | | | | | | |
| --- | --- | --- | --- | --- | --- | --- | --- | --- | --- | --- |
|  | **Pcrit cmH_2_O** | | | **pain VAS /10** | **lacrimation mm/s** | | **epworth** | | **snoring** | |
|  | baseline | 30 min | 48 hours | post | baseline | post | baseline | 48 hours | baseline | post |
| 1 | -15,1 | -15,4 | -35,9 | 0 | 19 | 9 | 5 | 4 | unknown | unknown |
| 2 | -26,6 | -23,7 | -30,5 | 0 | 59 | 53 | 8 | 7 | unknown | unknown |
| 3 | -46,5 | -44,8 | -21,7 | 0 | 0 | 9 | 12 | 13 | yes | unchanged |
| 4 | -92,6 | -30 | -70,4 | 1,5 | 42 | 48 | 4 | 4 | unknown | unknown |
| 5 | -12,9 | -15 | -15,6 | 0 | 40 | 26 | 3 | 3 | yes | increased |
| 6 | -25,2 | -15,1 | -23,3 | 0 | 34 | 11 | 9 | 8 | yes | unchanged |
| 7 | -27,5 | -26,5 | -25 | 0 | 80 | 70 | 6 | 8 | yes | unchanged |
| 8 |  |  |  | 1 | 6 | 22 | 6 | 3 | yes | unchanged |
| 9 |  |  |  | 3 | 50 | 42 | 20 | 15 | unknown | unknown |

| **patient** | **Active Manipulation** | | | | | | | | | |
| --- | --- | --- | --- | --- | --- | --- | --- | --- | --- | --- |
|  | **Pcrit cmH_2_O** | | | **pain VAS /10** | **lacrimation mm/s** | | **epworth** | | **snoring** | |
|  | baseline | 30 min | 48 hours | post | baseline | post | baseline | 48 hours | baseline | post |
| 1 | -12,5 | -16,7 | -21,7 | 6 | 9 | 21 | 4 | 1 | unknown | unknown |
| 2 | -56,8 | -54,6 | -97 | 8 | 35 | 46 | 6 | 7 | unknown | unknown |
| 3 | -32,2 | -36,7 | -33,7 | 8 | 33 | 16 | 20 | 15 | yes | reduced |
| 4 | -21 | -27,5 | -35,9 | 5 | 57 | 40 | 12 | 2 | unknown | unknown |
| 5 | -17,3 | -14,1 | -17 | 6,5 | 31 | 80 | 5 | 1 | yes | reduced |
| 6 | -20,1 | -33,4 | -22,1 | 8,5 | 22 | 50 | 16 | 11 | yes | reduced |
| 7 | -21,2 | -40,8 | -48,1 | 8 | 74 | 74 | 9 | 9 | yes | unchanged |
| 8 |  |  |  | 2 | 13 | 37 | 4 | 4 | yes | unchanged |
| 9 |  |  |  | 8 | 53 | 42 | 15 | 21 | yes | unchanged |
