# Supplementary material for: Upper airway stabilization by osteopathic manipulation of the sphenopalatine ganglion versus sham manipulation in OSAS patients: a proof-of-concept, randomized, crossover, double-blind, controlled study
Source: BMC Complement Altern Med. 2017 Dec 20;17:546. doi: 10.1186/s12906-017-2053-0 (PMC5738827; doi:10.1186/s12906-017-2053-0)
Supplement: Supplementary file 2 — “additional table: verbatim”. Patients’ verbatim descriptions after AM and SM, derivated from the sensation questionnaire. (DOCX 17 kb) [file 12906_2017_2053_MOESM2_ESM.docx]

Additional file 2

Additional table: verbatim

|  | Active manipulation |
| --- | --- |
| Patient 001 | Pain and spiritual well-being. |
| Patient 002 | Local and deep painful intrusion requiring relaxation, followed by a feeling of internal release and relaxation. |
| Patient 003 | Pain and unblocked nose. Feeling of flow in the mandible. |
| Patient 004 | Stabbing pain, impression of release of the jaw. Feeling of having more room in the mouth and a wider open mouth. Fatigue like after a long walk. |
| Patient 005 | Burning and scratching feeling on the jaw with a taste of blood. Feeling of circulating fluid associated with pain on the left side, “as if pressing on an abscess”. After the technique, increased tenderness but no pain of the first two incisors for 10 minutes. The operator’s explanations were reassuring despite the pain. |
| Patient 006 | Feeling of “relief” in the tonsils with the impression that the tonsils were elevated. Pain then feeling of firmer tonsils and less sensitive throat. Resolution of latent parieto-occipital headache present for 6 months after the technique, then feeling of having a straighter back. Pain resembling that of a bruise and an injection. |
| Patient 007 | Pain irradiating as electric shocks. |
| Patient 008 | Pain related to the headrest. |
| Patient 009 | Pain and prickling in the jaw. Feeling that something was blocked. Pain resembling that of an abscess. Subsequently, the right nostril and especially the left nostril became unblocked. Feeling of unblocked nose. |

|  | Sham manipulation |
| --- | --- |
| Patient 001 | Felt comfortable, impression that the technique will act as treatment and that it will be effective. |
| Patient 002 | Feeling of massage with vibrations with no particular effect. |
| Patient 003 | No particular feeling. |
| Patient 004 | Lacrimation and jaw popping. |
| Patient 005 | Non-painful massage of the gums. |
| Patient 006 | No particular symptoms apart from apprehension related to the proximity of a dental crown. Increased salivation. |
| Patient 007 | Non-painful vibration. |
| Patient 008 | Pressure on the gums. |
| Patient 009 | Nothing. |
